# Supplementary material for: Conformational ensemble-dependent lipid recognition and segregation by prenylated intrinsically disordered regions in small GTPases
Source: Commun Biol. 2023 Nov 2;6:1111. doi: 10.1038/s42003-023-05487-6 (PMC10622456; doi:10.1038/s42003-023-05487-6)
Supplement: Supplementary file 1 — Supplementary Material [file 42003_2023_5487_MOESM1_ESM.pdf]

*Supplementary Information*

# Conformational ensemble dependent lipid recognition and segregation by prenylated intrinsically disordered regions in small GTPases

Mussie K. Araya and Alemayehu A. Gorfe[*]

McGovern Medical School, University of Texas Health Science Center at Houston, Department of Integrative Biology and Pharmacology, 6431 Fannin St., Houston, Texas 77030

[*]Corresponding author: Alemayehu.G.Abebe@uth.tmc.edu

**Keywords:** Intrinsically disordered region; membrane; polybasic domain; lipid-protein interaction; membrane asymmetry

**Supplementary Table 1: Summary of non-standard abbreviations used in the main text.**

| Abbreviation | Definition | Remarks |
|---|---|---|
| PIDR | Prenylated intrinsically disordered region | Common in lipid modified small GTPases |
| PM | Plasma membrane | |
| Farn (also called SYSF) | Farnesyl | Found in Rheb |
| GG (also called SYSG) | Geranylgeranyl | Found in RhoA, Rap1A, Rap1B, Rac1, CDC42b |
| Palm (also called CYSP) | Palmitoyl | Found in Rac1 |
| PBD | Polybasic domain | Found in RhoA, Rap1A, Rap1B, Rac1, CDC42b |
| Np | Number of basic residues | |
| K:R | Lys to Arg ratio | |
| Nh | The ratio of hydrophobic to total number of residues | |
| B:A | The ratio of basic to acidic residues | |
| *S* | Spacing between the last PBD residue and the prenylated Cys | |
| RMSD | Root mean square deviation | |
| Rg | Radius of gyration | |
| RC | Reaction coordinate | |
| APL | Area per lipid | Measured using the pure-POPC monolayer water box lateral dimensions. |
| P-P | Head-to-head distance | Measured as the distance between the average phosphorous atom z-positions in the two leaflets of a bilayer. |
| HB | Hydrogen bond | |
| $N_{HB}$ | Number of hydrogen bonds | |
| $N_C$ | Number of hydrophobic (carbon-carbon) contacts | |

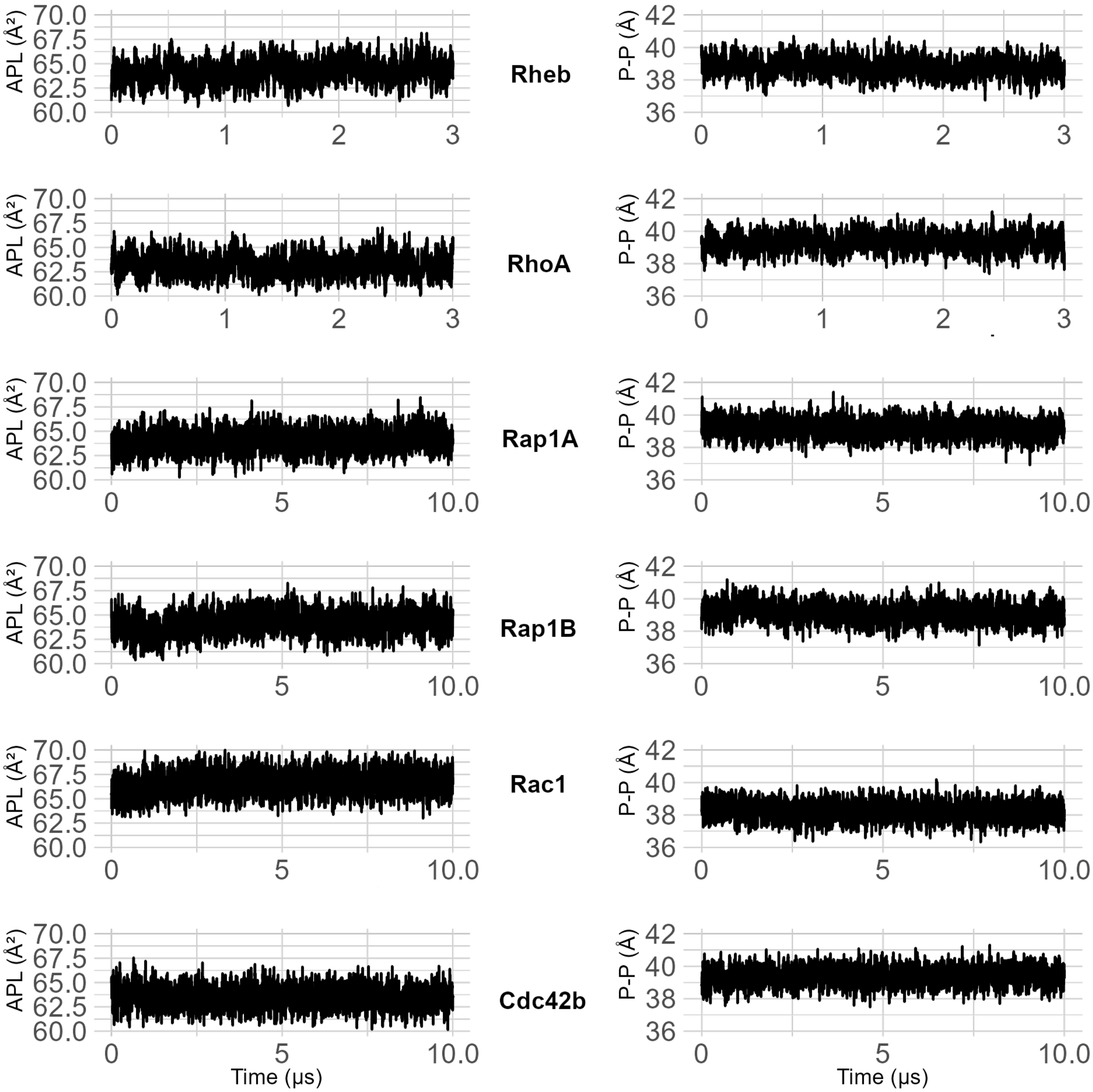


**Supplementary Figure 1.** Time evolution of area per lipid (APL) and bilayer thickness (P-P) for each of the Rheb, RhoA, Rap1A, Rap1B, Rac1 and Cdc42b PIDR simulations. APL was calculated using the simulation box lateral dimensions at the pure-POPC monolayer. P-P was measured as the average distance along the membrane normal between phosphorous atoms at the two leaflets. The Rheb and RhoA simulations were 3µs long and the rest were10µs.

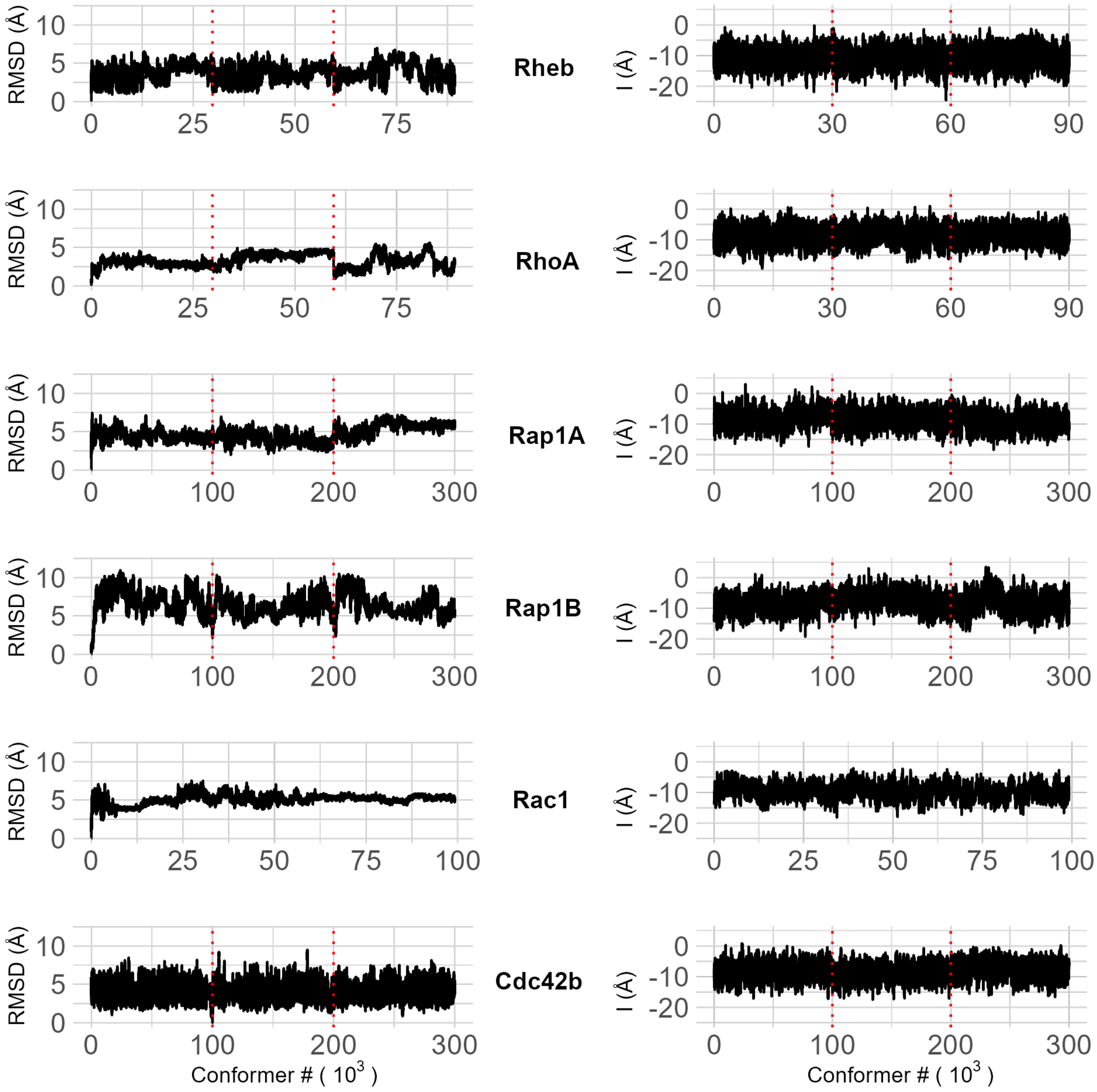


**Supplementary Figure 2.** Root-mean-square deviation (RMSD; left) and membrane insertion depth (I; right) of the simulated PIDR peptides. RMSD was measured using the initial structures as reference, and I was measured using the center-of-mass of the prenyl chain (z-distance of the center-of-mass of the prenyl chain from the bilayer center). Vertical dotted lines demarcate the three peptides in each simulation except Rac1 where only one peptide was considered (see main text for explanation). Trajectories were sampled every 100ps.

A

| Structural element | Rheb | RhoA | Rap1A | Rap1B | Rac1 | Cdc42b |
|---|---|---|---|---|---|---|
| β-bridge | 0.2 | 0.0 | 0.1 | 0.4 | 1.0 | 0.8 |
| Extended | 0.0 | 0.0 | 0.0 | 0.0 | 0.0 | 3.7 |
| Coil | 58 | 78 | 60 | 63 | 62 | 83 |
| Turn | 40 | 21 | 31 | 33 | 37 | 11 |
| $3_{10}$-helix | 1.6 | 0.1 | 6.1 | 2.0 | 0.0 | 0.4 |
| α-helix | 0.0 | 0.0 | 2.9 | 1.0 | 0.0 | 0.8 |

B

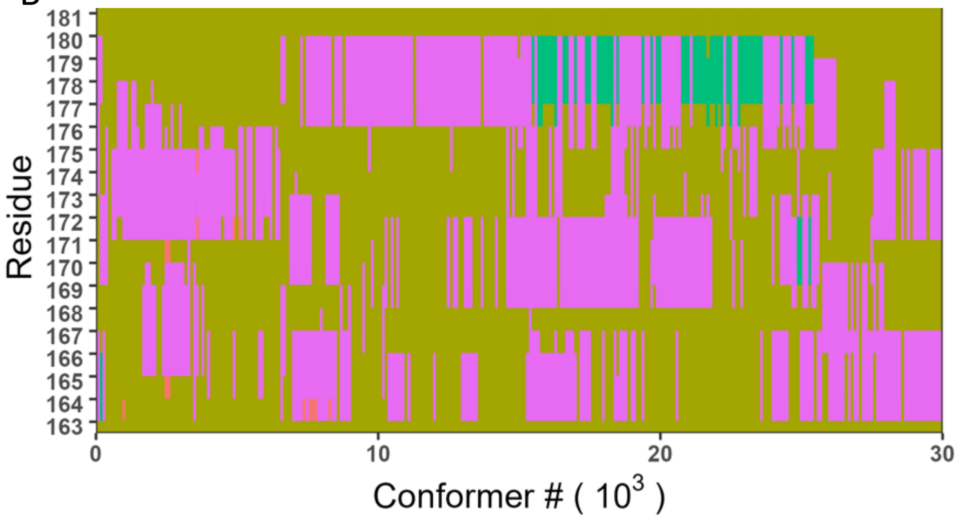


**Supplementary Figure 3**. The PIDRs remained unstructured during the simulations. (**A**) Percentage of backbone secondary structure elements sampled during the simulation of each PIDR, showing that the simulated conformers are predominantly unstructured (random coil and turn). (**B**) Time evolution of secondary structure elements using Rap1A as an example (color code as in panel **A**), showing that the peptide fluctuates between random coil and turn with rare transitions to helical conformations (sampling frequency was 1ns and concatenated data for the three peptides was used).

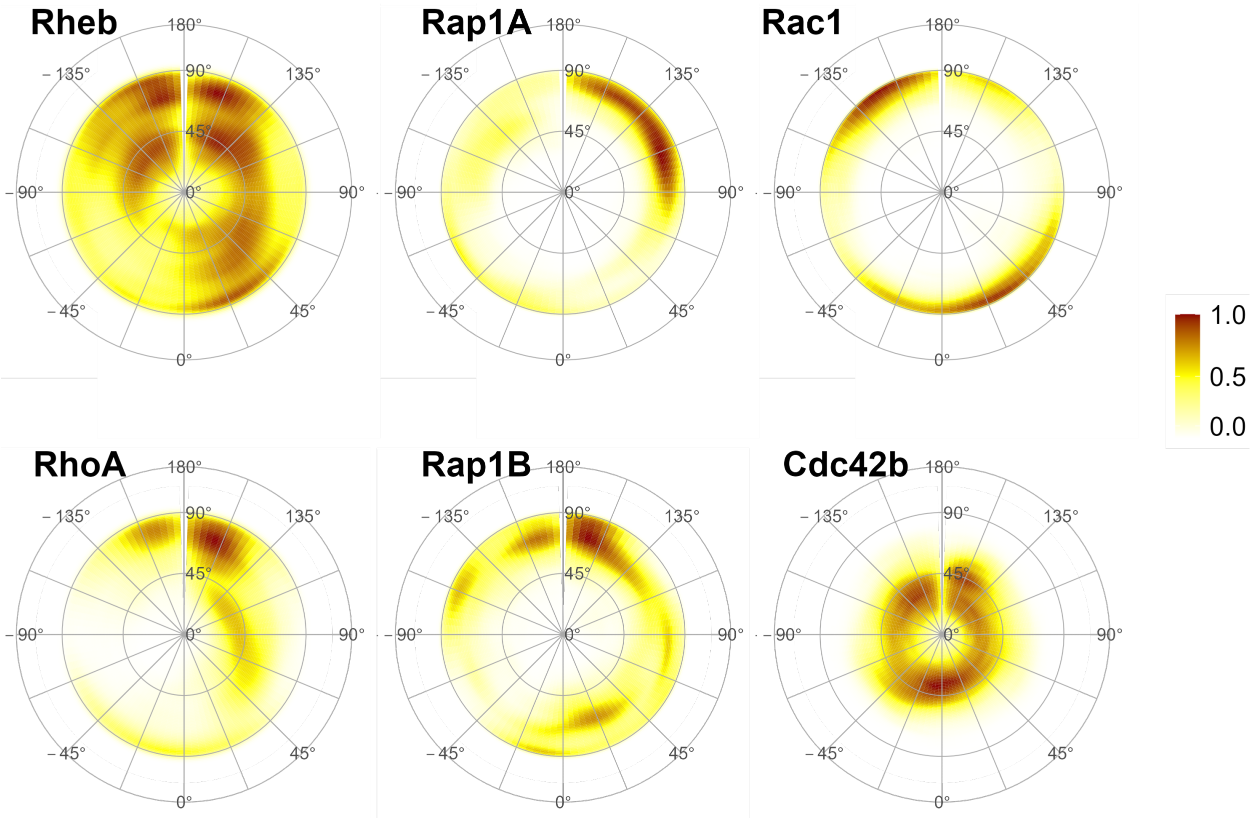


**Supplementary Figure 4**. Normalized density maps of backbone tilt and rotation angles with the tilt angle [0, 90] shown along the radial coordinate and rotation angle along the angular coordinate [−180, 180], quantifying the orientation of each PIDR relative to the bilayer normal.

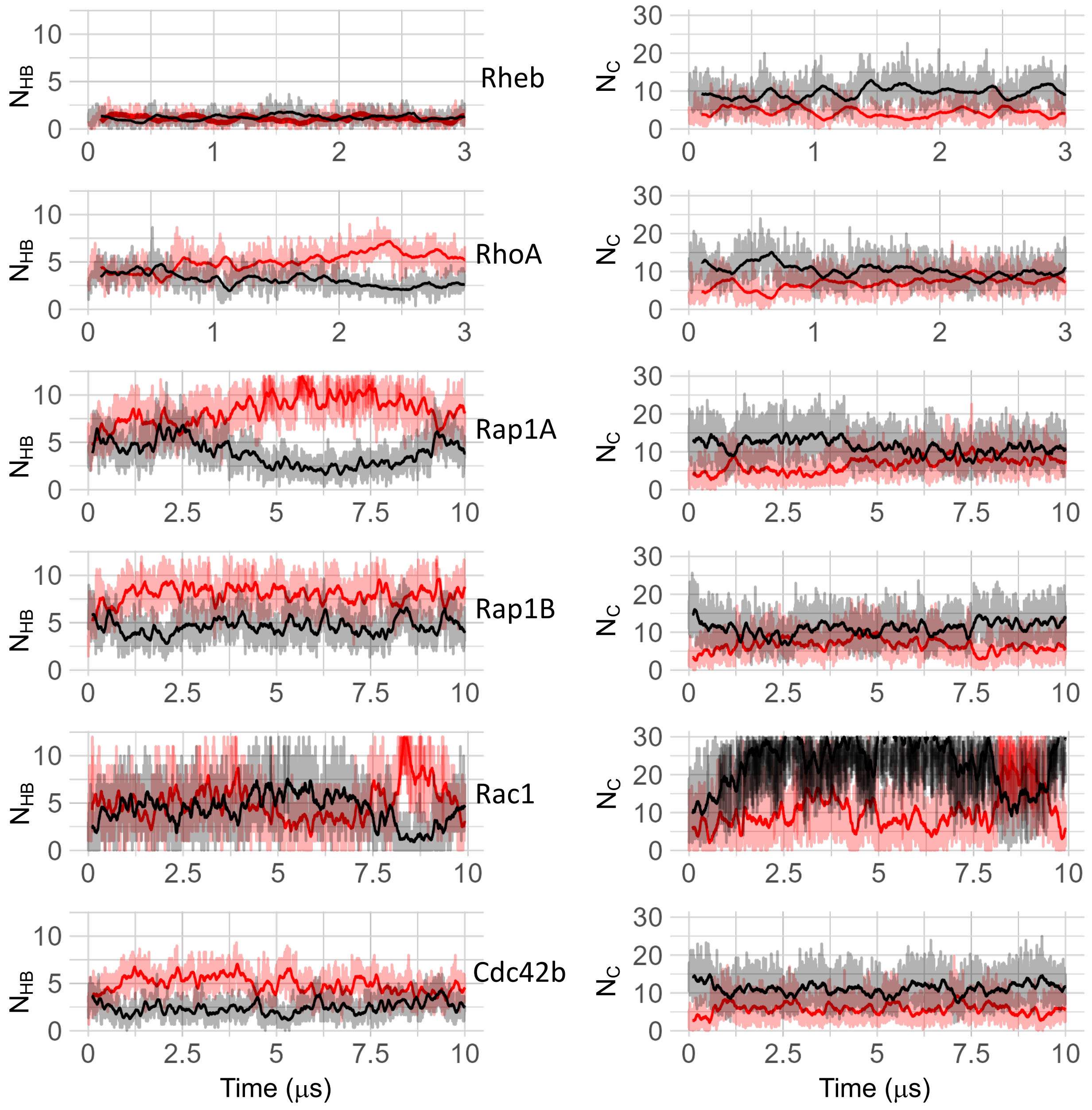


**Supplementary Figure 5.** Time series of numbers of hydrogen bonds per peptide ($N_{HB}$, left panels) and vdW contacts per peptide ($N_C$, right panels) with PS (red) and PC (black) lipids. HB was calculated using all charged and polar sidechains and lipid headgroup oxygen atoms, and $N_C$ using carbon atoms of non-polar sidechains (excluding proline) and lipid acyl chains. Data sampled every 1ns is shown in lighter shades and 100ns running averages in darker shades.

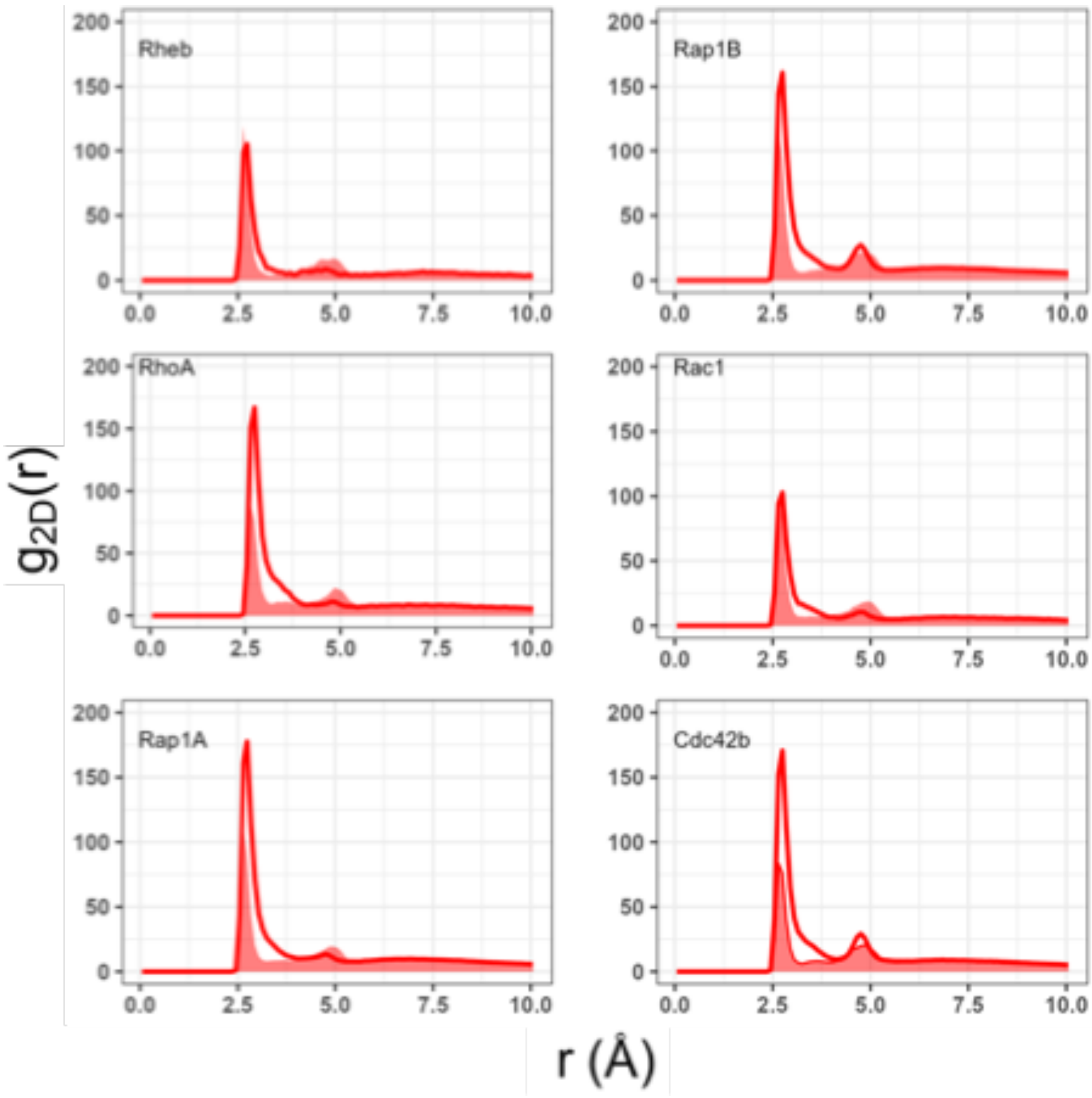


**Supplementary Figure 6.** Two-dimensional radial pair distribution of POPS carboxyl (lines) and phosphate (shaded) oxygen atoms around Lys and Arg side chain nitrogen atoms of each PIDR. Both oxygen atoms of the serine headgroup (O13A and O13B in CHARMM), the two phosphate oxygen atoms that are not bonded with carbon (O13 and O14), one of the terminal nitrogen atoms of Arg (NH1), and Nz of Lys were used for the calculations.
