# Supplementary material for: Conformational ensemble-dependent lipid recognition and segregation by prenylated intrinsically disordered regions in small GTPases
Source: Commun Biol. 2023 Nov 2;6:1111. doi: 10.1038/s42003-023-05487-6 (PMC10622456; doi:10.1038/s42003-023-05487-6)
Supplement: Supplementary file 2 — Description of Additional Supplementary Files [file 42003_2023_5487_MOESM2_ESM.pdf]

Description of Additional Supplementary Files

File name: Supplementary Data 1
Description: The source data behind Figure 2 in the paper.

File name: Supplementary Data 2
Description: The source data behind Figure 3 in the paper.

File name: Supplementary Data 3
Description: The source data behind Figure 5 in the paper.

File name: Supplementary Data 4
Description: The source data behind Figure 6 in the paper.

File name: Supplementary Data 5
Description: The source data behind Figure 7 in the paper.

File name: Supplementary Data 6
Description: The source data behind Figure 8 in the paper.
